# Cell Surface Display of Poly(3-hydroxybutyrate) Depolymerase and its Application

**DOI:** 10.4014/jmb.2001.01042

**Published:** 2020-02-10

**Authors:** Seung Hwan Lee, Sang Yup Lee

**Affiliations:** 1 Department of Biotechnology and Bioengineering, Chonnam National University, Gwangju 686, Republic of Korea; 2 Metabolic and Biomolecular Engineering National Research Laboratory, Department of Chemical and Biomolecular Engineering (BK1 Program), Institute of BioCentury, Korea Advanced Institute of Science and Technology, Daejeon 34141, Republic of Korea

**Keywords:** Cell surface display, depolymerase, immobilization, enantioselective biocatalyst, OprF

## Abstract

We have expressed extracellular poly(3-hydroxybutyrate) (PHB) depolymerase of *Ralstonia pickettii* T1 on the *Escherichia coli* surface using *Pseudomonas* OprF protein as a fusion partner by C-terminal deletion-fusion strategy. Surface display of depolymerase was confirmed by flow cytometry, immunofluorescence microscopy and whole cell hydrolase activity. For the application, depolymerase was used as an immobilized catalyst of enantioselective hydrolysis reaction for the first time. After 48 h, (*R*)-methyl mandelate was completely hydrolyzed, and (*S*)-mandelic acid was produced with over 99% enantiomeric excess. Our findings suggest that surface displayed depolymerase on *E. coli* can be used as an enantioselective biocatalyst.

Polyhydroxyalkanoates (PHAs) are biodegradable polyesters, that accumulate intracellularly as a carbon and/or energy source in numerous bacteria, to provide renewable resources under nutrient limited conditions. In PHA-accumulating bacteria, many enzymes, including PHA synthase, phasin, epimerase, oligomer hydrolase and depolymerase, play a role in the biosynthesis and degradation of PHAs [1-4]. Of those, PHA depolymerases have drawn much attention for their novel characteristics such as high stability, relatively small molecular weight (< 70 kDa), consisting of only one polypeptide, and strong affinity to hydrophobic materials [5]. Some bacteria secrete depoly-merases to degrade extracellular PHAs and utilize the resulting monomer, 3-hydroxyalkanoic acid, as a nutrient. Several researchers have begun to employ depolymerases as enantioselective catalysts for biotransformation, and selective binding in immunoassays [6, 7].

Thus far, various surface display systems have been reported, with potential applications in the development of vaccines, bioadsorbents, biocatalysts, antibody libraries and biosensors [8-10]. In addition, many researchers have demonstrated the use of enzymes as target proteins for cell surface display due to their unique characteristics, such as chemo-, regio- and enantioselectivity [11,12]. Among the numerous available enzymes only a few have been adapted for cell surface display applications, such as lipase and organophosphorus hydrolase for biocatalysts and bioreme-diation, respectively [13-15]. Therefore, the development of new useful enzymes for display systems is an important research objective. Recently, a Japanese research group successfully applied cell surface display of PHB depolymerase, however, the application was limited to the degradation of the polyester to produce 3-hydroxybutyrate [16, 17]. In this paper, we report the display of the *Ralstonia pickettii* T1 depolymerase on the surface of *E. coli* cells using truncated *Pseudomonas* OprF as an anchoring motif and demonstrate the biocatalytic application of this system to enantioselective hydrolysis of a racemic ester.

To construct pTacOprF164RD and pTacOprF188RD, *R. pickettii* T1 depolymerase gene was amplified using primers 5’-gctctagagccacggcggggcccggtgc-3’ and 5-cccaagctttcatgg acaattgccgacg-3’, and inserted into the XbaI and HindIII sites of pTacOprF164PL and pTacOprF188PL, respectively. For the immunodetection, 6 histidine residues (underlined) were added to C-terminal of depolymerase gene, which was amplified using primers 5’-gctctagagccacggcggggcccggtgc-3’ and 5’-cccaagctttcaatggtgatgatggtgatgtggacaattgccgacg-3’, and cloned into the XbaI and HindIII sites of pTacOprF164PL and pTacOprF188PL to make pTacOprF164RDH and pTacOprF188RDH, respectively. Primers for the amplification of the *R. pickettii* T1 PHB depolymerase gene were designed based on the reported sequence (J04223) [18]. Plasmids used in this study are listed in Table 1.

For detection of displayed depolymerase fused with 6 histidine residues, recombinant cells were labeled with mouse anti-His antibody (Sigma, USA) diluted 1:100, followed by rabbit anti-mouse IgG antibody conjugated fluorescein isothiocyanate (FITC) diluted 1:200. The cells were analyzed using a flow cytometer (FACSCalibur, Becton Dickinson, USA). For the visualization under fluorescence microscope, cells were stained with the mouse anti-His antibody diluted 1:1000 and rabbit anti-mouse IgG conjugated with FITC diluted 1:3000. The samples were visualized by confocal fluorescence microscopy (Zeiss LSM 410, Carl Zeiss, Germany).

After collection by centrifugation at 5,590 ×g and 4°C and washing with distilled water, recombinant cells were further freeze-dried for 48 h with lyophilizer (TFD5505, Ilshin Lab., Korea) for measuring whole cell enzyme activity and application of enantioselective biocatalyst. Hydrolytic activity of genetically immobilized depolymerase was assayed by spectrophotometric method as mentioned elsewhere [14]. In order to observe the enantioselective catalytic property of cell surface displayed depolymerase (Fig. 1), 300 mg of freeze-dried XL10-Gold (pTacO188RD) and 150 mg of racemic methyl mandelate (Sigma) were added to 30 ml of 50 mM Tris buffer (pH 8.0). Reaction mixture was maintained at 250 rpm at 37oC. Quantification of chemicals were determined by HPLC as previously described [19].

To date, cell surface display systems have employed many enzymes including lipase, polyethylene terephthalate (PET) degrading enzyme (PETase), cyotochrom P450, amylase, dimeric bovine adrenodoxin and carboxymethylcellulase as target proteins [11, 12, 20, 21]. As we mentioned earlier, PHB depolymerases have ideal characteristics for cell surface display applications. *P. aeruginosa* PaO1 OprF was selected for depolymerase display as it allows successful and stable display of proteins in an active form on the surface of *E. coli* and *P. putida* [14, 22].

First, surface display of the depolymerase was examined by flow cytometer analysis. Fluorescence was analyzed for *E. coli* (pTacOprF188E) and *E. coli* (pTacOprF188RDH) incubated with anti-His antibody followed by FITC-conjugated secondary antibody. As shown in Fig. 2A, the mean fluorescence values of XL 10-Gold harboring pTacOprF188RDH was increased when compared to that of XL 10-Gold harboring pTacOprF188E indicating that the fusion protein of truncated OprF (OprF_t_) and depolymerase, with 6 histidine residues, was expressed outside of the *E. coli*. In order to confirm surface display of depolymerase, recombinant cells were analyzed using immunofluorescence microscopy. *E. coli* XL-10 Gold (pTacOprF188RDH) labeled with anti-His antibody followed by binding of FITC-conjugated secondary antibody showed fluorescent spots, which demonstrates depolymerase fused with 6 histidine residues was successfully expressed on the cell surface (Fig.2C). Meanwhile, *E. coli* XL-10 Gold cells harboring pTacOprF188E showed little fluorescence (Fig. 2B).

After confirmation of surface display, we further checked whole cell hydrolase activity to ascertain functional expression of depolymerase. After normalizing hydrolase activity for recombinant XL 10-Gold (pTacOprF188RD) obtained with 0.1 mM IPTG induction to a value of 100, the relative activities with 0.01 and 1 mM IPTG were 21.3 and 64.8, respectively. Little hydrolase activity was observed in supernatant fractions. These suggest that OprF_t_ successfully played the role of an anchoring motif for the surface display of depolymerase on the *E. coli* surface in an active form, without protein secretion and significant cell lysis.

For application in biocatalysts, the enantioselective resolution of racemic methyl mandelate was investigated. The scheme of this reaction is shown in Fig. 1. Time profiles of enantiomeric excess of product during enantioselective resolution are shown in Fig. 3. The conversion of the reaction and enantiomeric excess of the product, (*S*)-mandelic acid, obtained in 48 h were over 40% and 99%, respectively.

It is known that PHA depolymerase shows enantioselective behavior towards substrate in aqueous solutions, however, the main application of surface displayed depolymerase has been the hydrolysis of polymer or oligomer, composed of monomeric units of 3-hydroxyacid [5, 16, 17]. Here, we have shown for the first time, that cell surface displayed depolymerase can enantioselectively hydrolyze not only polymers or oligomers of PHB, but racemic esters. As shown in Fig. 3, depolymerase displayed on the cell surface exhibited good enzymatic characteristics, such as high enantiomeric excess and conversion as an enantioselective catalyst. It is clear that depolymerase is stably expressed at the outer membrane as an active form, which is very similar to an immobilized form of enzyme without elimination of selectivity and activity. These suggest that *E. coli* cells displaying depolymerase have potential as industrial catalysts similar to other hydrolases.

In conclusion, we reported the cell surface display of PHB depolymerase on the cell surface of *E. coli* and demonstrate the application of this system to the enantioselective biotransformation for the first time. Furthermore, improvement of the enzyme characteristics via methods such as immobilization, fusion technique and directed evolution, could result in significant increases in activity, selective binding affinity or other key characteristics of depolymerases. Thus, the results of our efforts can be successfully applied to the various fields including bioremediation, biocatalysis, immunoassay, whole cell microarrays and whole cell biosensors.

## Figures and Tables

**Fig. 1:**
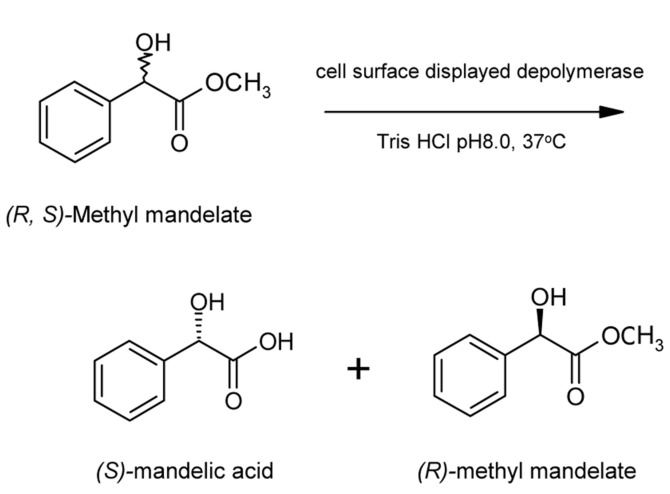
Reaction scheme for the enantioselective hydrolysis of racemic methyl mandelic acid.

**Fig. 2:**
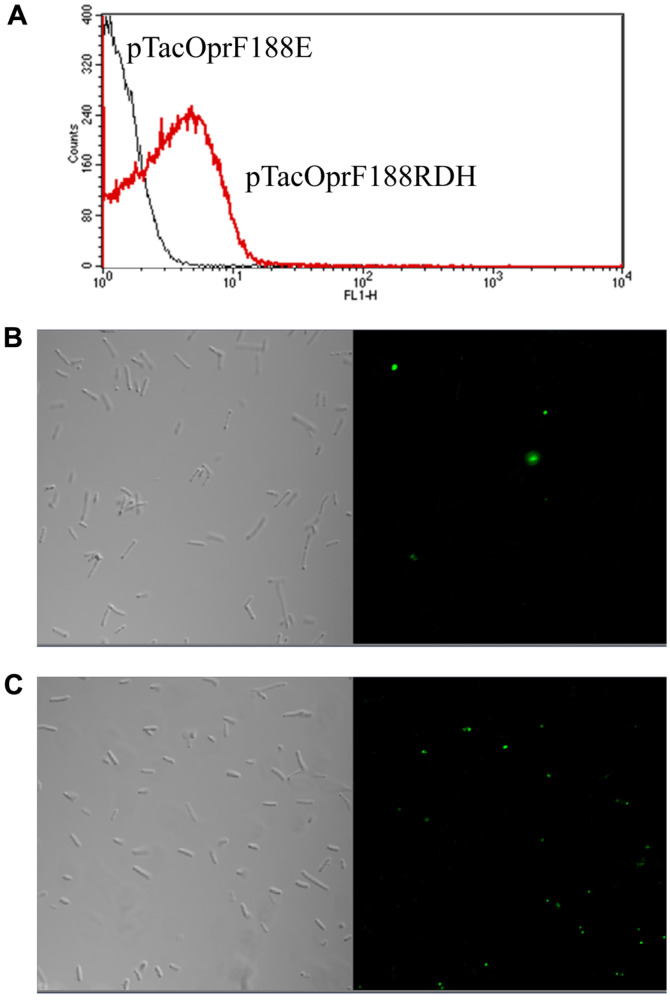
Flow cytometry analysis (**A**) of *E. coli* XL-10 Gold harboring pTacOprF188E and pTacOprF188RDH. Differential interference micrographs (left) and immunofluorescence micrographs (right) of *E. coli* XL-10 Gold harboring pTacOprF188E (**B**) and pTacOprF188RDH (**C**). Cells were incubated with mouse anti-His probe antibody followed by probing with rabbit anti-mouse IgG-FITC conjugate.

**Fig. 3:**
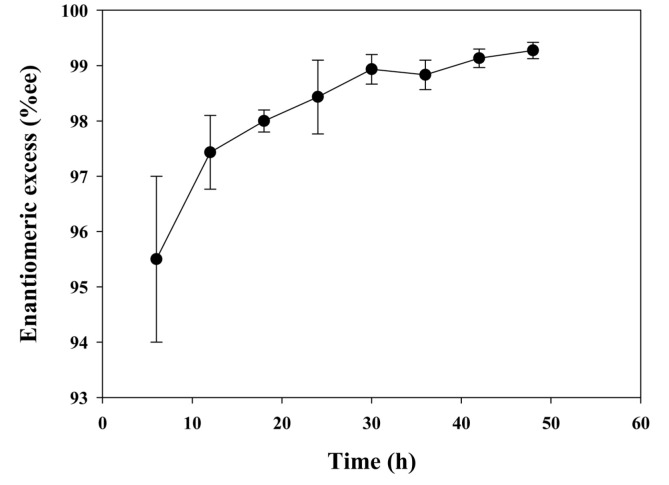
Time profiles for the enantioselective resolution of racemic methyl mandelic acid using cell surface displayed depolymerase. Time profiles of enantiomeric excess (●) of reaction products are shown.

**Table 1:** List of plasmids used in this study.

Plasmid	Relevant Characteristics	Reference or Source
pTacOprF164PL	pTacOprF164 derivative;	[14]
	*P. fluorescens* SIK W1 lipase gene	
pTacOprF188PL	pTacOprF188 derivative;	[14]
	*P. fluorescens* SIK W1 lipase gene	
pTacOprF188E	pTac99A derivative; containing 636 bp fragment of *oprF* of *P. aeruginosa* and the stop codon	[14]
pTacOprF164RD	*R. pickettii* T1 depolymerase gene	This study
pTacOprF164RDH	*R. pickettii* T1 depolymerase gene with 6 histidine residues	This study
pTacOprF188RD	*R. pickettii* T1 depolymerase gene	This study
pTacOprF188RDH	*R. pickettii* T1 depolymerase gene with 6 histidine residues	This study
